# Examining the association between perceived stigma, its correlates, and restrictions in participation among persons with disabilities in Nepal: a cross-sectional study

**DOI:** 10.1186/s12889-024-18682-9

**Published:** 2024-04-26

**Authors:** Hridaya Raj Devkota, Sasmita Poudel, Mohan Krishna Shrestha, Radhika Upreti Oli, Nabin Kumar Rai, Manish Poudel, Pradeep Banjara, Catherine Malla, Yadira Perez Hazel, Anjila Dahal, Reeta Gurung

**Affiliations:** 1 Institute for Social and Environmental Research Nepal (ISER-N), Bharatpur-15, Chitwan, Nepal; 2 Institute of Himalayan Risk Reduction, Kathmandu, Nepal; 3 Tilganga Institute of Ophthalmology, Kathmandu, Nepal; 4 The Fred Hollows Foundation, Sydney, Australia

**Keywords:** Disability, Perceived stigma, Self-stigmatization, Correlates, Participation, Nepal

## Abstract

**Background:**

Disability stigma in low- and middle-income countries is one of the most persistent and complex barriers limiting persons with disabilities (PwDs) from enjoying their rights and opportunities. Perceived stigma among PwDs and its impact on participation restriction is rarely assessed in Nepal.

**Objective:**

This study aimed to measure the extent of perceived stigma by PwDs, identify its relationships with specific demographic factors, and assess the impact on social participation.

**Methods:**

A cross-sectional survey was conducted between May and July 2022 among PwDs in Nepal, with a sample of 371. The Explanatory Model Interview Catalog (EMIC) stigma scale and P-scale suitable for people affected by stigmatized conditions were used, and the generated scores were analyzed. One-way ANOVA was performed to determine group differences for sociodemographic variables, and linear regression and correlational analysis were used to identify their association and measure the strength and direction of the relationship.

**Results:**

The mean stigma score was 16.9 (SD 13.8). 42% of respondents scored higher than the mean. The scores differed significantly by disability type, caste and ethnicity, education, occupation, and household wealth. Over 56% reported participation restriction, and 38% had severe/extreme restriction. Approximately 65% of participants with intellectual disabilities, 53% with multiple disabilities, and 48.5% of persons with severe or profound disabilities experienced severe or extreme restrictions. Perceived stigma had a positive correlation with Disability type (*r* = 0.17, *P* < 0.01) and negative correlations with Severity of disability (*r*= -0.15, *P* < 0.05), and Household wealth (*r*= -0.15, *P* < 0.01). Education was inversely associated with both stigma (*r*= -0.24, *P* < 0.01), and participation restriction (β= -9.34, *P* < 0.01). However, there was no association between stigma and participation restriction (β= -0.10, *P* > 0.05).

**Conclusion:**

All participants exhibited stigma in general; however, the severity varied based on disability type, level of education, and sociocultural circumstances. A large proportion of participants reported facing a high degree of restrictions in participation; however, no association was detected between perceived stigma and participation restriction. A significant negative linear correlation was observed between education and participation restriction. Stigma reduction programs focusing on education and empowerment would be especially important for overcoming internalized stigma and increasing the participation of PwDs.

## Background

Persons with disabilities are those who have long-term physical, mental, intellectual or sensory impairments, which in interaction with various barriers may hinder their full and effective participation in society on an equal basis with others [1]. Stigmatizing attitudes and beliefs toward disability have been regarded as the most prevalent and complex obstacle preventing persons with disabilities from accessing their basic rights and opportunities, including health care, education, employment, and social participation [2, 3]. The detrimental impact of disability stigma on individual life is widely acknowledged. In the academic literature, stigma is described inconsistently. According to Goffman and Pescosolido, stigma is an attribute that is deeply discrediting and as a mark separating individuals from one another based on a socially conferred judgment that some persons or groups are tainted and “less than”. Stigma often leads to negative beliefs, stereotypes, prejudice, and a desire to avoid or exclude stigmatized persons [4]. The literature suggests that stigma is related to problems of knowledge, attitudes, and behavior [5]. Stigma exhibits a range of feelings, beliefs, and attitudes, shown or experienced behaviors and practices as lived realities. Thus, stigma is categorized as felt (perceived or anticipated) stigma, enacted (experienced) stigma, and internalized or self-stigma [6, 7].

Perceived or enacted public stigma, and felt or internalized self-stigma are two separate constructs [8]. The theory of self-stigma suggests that negative social judgments lead to worse outcomes through internalization processes that apply stigmatizing social beliefs to oneself [9]. Modified labeling theory developed by Link and colleagues suggests that stigmatized individuals are likely to have internalized sociocultural conceptions that become self-relevant, and the individual anticipates devaluation, discrimination, and rejection due to stigma, which, in turn, leads them to increased social isolation and decreased employment, leisure and social opportunities that may result in decreased self-efficacy, self-esteem and self-worth by the individual [10]. Additionally, the study revealed that self-stigma acts as a more destructive stressor than public stigma [11].

Poor understanding and lack of awareness regarding the causes of disabilities, along with prevalent misconceptions and myths regarding the cause of disabilities and their resulting consequences, have perpetuated the notion that disabilities are often attributed to misdeeds and wrongful acts in past life. The existing body of literature frequently highlights contextual factors, such as traditional, cultural, or religious beliefs, as significant contributors to the stigmatization of disabilities [3, 12]. Studies in Nepal found that myths, folklore and misconceptions in culture, tradition and religion about disability are deeply rooted and often cited as the basis for individual beliefs and attitudes. These beliefs come from many traditions, including Hinduism, Buddhism and Islam [13]. In many settings, disability-related stigma was also instigated by feelings of social dislike, largely because of associations of impairments with wrongdoings, witchcraft, or punishment by God. Study findings further suggest that these misconceptions are often reinforced by insensitive media coverage that perpetuates negative perspectives and contributes to the persistence of disability-related stigma [14].

The literature commonly reports that place of residence, age, socioeconomic status, severity, and type of disability are associated with stigma. A study in India reported moderate to high levels of internalized stigma among people with bipolar disorder [15]. Younger generations, those with lower socioeconomic status, and severely impaired people are found to have high levels of internalized stigma, while rural residents are found to have high levels of enacted stigma [16]. A study in Ghana among stroke survivors showed no association between rural/urban location and internalized stigma [17]. Social factors such as gender in many contexts are found to be linked to higher levels of stigma and discriminatory practices against girls and women with disabilities [13, 18].

Erving Goffman developed a theory of stigma centered on social interaction and exclusion and the notion of deviance [19]. In line with this, some studies conducted in developing countries have revealed consequences of stigma from psychosocial dysfunction to isolation, rejection, and participation restriction [3] and high-level feelings of alienation and social withdrawal [20].

Most studies conducted in developing countries that identify the factors shaping stereotypes and stigma surrounding disabilities have particularly focused on the perspective of the public without disabilities and rarely studied the construction and consequences of self-stigma among persons with disabilities [21, 22]. The studies also uncovered a broad spectrum of discriminatory behaviors and practices faced by persons with disabilities, stemming from the stigma that exists at various levels, including within their own families, communities, and the wider society. This study aimed to assess the extent of stigma perceived by persons with disabilities, its relationships with sociodemographic factors, and the impact on social participation. Specifically, the study measures the degree of perceived stigma among persons with disabilities, explores the associations between stigma and sociodemographic and other factors, and assesses the impact of perceived stigma on the participation restriction of persons with disabilities in Nepal. The study is expected to contribute to filling the existing knowledge gaps in this field and provide valuable insights that can assist policy planners in formulating strategies to reduce stigma and enhance social participation opportunities for persons with disabilities in Nepal.

## Methodology

### Study setting

The study was conducted in an eye health project districts – Bara, Rautahat and Makawanpur, implemented by Tilganga Institute of Ophthalmology, Kathmandu. The data was collected from seven municipalities of Bara, Rautahat and Makawanpur districts, located in the mid-southern part of Nepal with a population of 99,930 covering 72 wards of seven municipalities. The study municipalities have a diverse population in terms of culture, religion, caste and ethnicity. The predominant religion in the area is Hinduism, followed by Buddhism and Islam. The population settlements in the study area comprised a mixture of hill migrants, indigenous (tribal) and Tarai-Madhesi populations. Notably, the privileged caste groups, namely, Brahmins, Chhetris, and Newars, constitute a minority. The national census 2021 reported disability rates of 2.19%, 1.41% and 1.33% of the total population in Makawanpur, Bara and Rautahat districts, respectively, while the national average was 2.25% [23].

### Study design

This study was part of a larger project and was conducted between May and July 2022. The study was a cross-sectional survey that included men and women with disabilities who were 18 years or older and living in the project districts. The data presented in this study is a subset of the data collected during the larger project implementation and was collected by the same authors.

### Participant identification and recruitment

The participants were identified with the help of local organizations of persons with disabilities (OPDs), and snowballing method. Adopting a complete enumeration technique, the participants were recruited developing some criteria. The inclusion criteria for recruitment were: an age of 18 years or above and a resident of the study area, having cognitive ability to comply with the instructions and respond to the questions, able to communicate verbally or using sign language, possessing a disability ID card issued by the government and/or defined as having a disability by the Washington Group disability criteria (short set). Out of 371 identified persons with disabilities who met the inclusion criteria and were approached for an interview, one individual declined to participate, resulting in a total of 370 participants for recruitment in the study.

### Survey instrument and data collection procedure

A semi structured sociodemographic questionnaire along with two scales, a 15-item stigma scale (EMIC stigma scale) and an 18-item participation scale (P-Scale), both interview-based instruments for measuring the level of perceived stigma and participation restriction, respectively, were used. The sociodemographic information collected included participants’ place of residence, gender, age, caste and ethnicity, religion, education, occupation, marital status, disability type, and household wealth.

The Explanatory Model Interview Catalog (EMIC) stigma scale was originally designed to measure stigma among leprosy-affected people and later adapted for people with disability.

It measures each question with four options, “yes,” “possibly,” “uncertain,” and “no.” Scores were generated by assigning 3 points to “yes,” 2 to “possibly,” 1 to “uncertain,” and 0 to “no” for all questions except question 2, in which a reverse scoring method was employed.

The P-scale is generic and particularly suitable for use among people affected by stigmatized conditions, such as leprosy, disability, and HIV/AIDS. It measures how respondents rate their participation in comparison with a ‘peer’, defined as ‘someone similar to the respondent in all aspects except for the disease or disability’ [24]. The scale is based on the participation domain of the international classification of functioning (ICF), which includes nine domains: learning and applying knowledge, general tasks and demands, communication, mobility, self-care, domestic life, interpersonal interactions and relationships, major life areas and community, and social and civic life. When respondents reported restriction in a specific area (“no” or “sometimes”), they were asked to indicate the level of restriction: 1 - no problem, 2 - a small problem, 3 - a medium problem, and 5 - a large problem. The sum of scores was calculated, with a higher total score representing a lower level of participation. Both scales, the EMIC stigma scale and the P-scale, have already been validated and widely used in Asian countries, including Nepal [25, 26]. The internal consistency of the EMIC stigma scale and P-scale was confirmed with a Cronbach’s alpha coefficient of 0.89 and 0.96 respectively.

All tools – the questionnaire, EMIC stigma scale, and P-scale - were set up on tablet computers and mobile phones with KoBo Collect software and were administered through face-to-face interviews in Nepali language by trained field researchers. The questionnaire was first developed in English, then translated into Nepali by three bilingual Nepalese translators and subsequently field-tested for acceptability and comprehension among the target population. Similarly, the P-scale and EMIC stigma scale were also translated from English to Nepali following the guidelines stated by the authors [27]. A bilingual translator performed a back translation to English. Before administration, all Nepali versions of the tools were field tested, and minor amendments were made to ensure readability.

Two supervisors and eight enumerators with prior experience in research data collection were given a 3-day training before being sent to the field to familiarize them with study tools, data collection techniques, and KoBo Collect. A local sign language interpreter and the family members (in some cases) helped to interview participants with hearing impairment.

### Measures

The P-scale score was the outcome variable that ranged from 0 to 90. In the P-scale, the cutoff (threshold) score for ‘normal’ (= not having significant participation restrictions) was determined at 12. People scoring more than 12 were classified as having participation restrictions. The severity levels were categorized as mild (13–22), moderate (23–32), severe (33–52), and extreme restriction (53–90) [28].

The covariate stigma score ranged from 0 to 43. We obtained a composite score for each respondent by summing the scores of the 15 questions. A higher score implied a higher level of perceived or experienced stigma faced by the respondent. A composite index was developed for household wealth summing up the rated scores for the selected household items, and the score was recoded and ranked into quintiles. This study interpreted the correlation criteria with r values of 0–0.25 = a weak correlation, 0.25–0.5 = fair correlation, 0.5–0.75 = moderate correlation, and > 0.75 = strong correlation [29].

Table 1 presents the variables and their definitions used in the study.

**Table 1:** Variables and their definitions used in the study

Variables	Definition
**Outcome variable**	
P-scale score	Total algebraic sum of the rated scores by the respondents (between 0–90)
**Covariate**	
Stigma Score	Total algebraic sum of the rated scores by the participants (between 0–43)
**Sociodemographic variables**	
Place of residence	Respondent’s place of residence – rural or urban. Respondents living in Rural Municipalities at the time of the survey are categorized as rural and those living in Urban Municipality categorized as urban residents.
Gender	Self-reported sex identity of respondent - male or female
Age	Completed age in years of respondent at the time of survey
Caste and ethnicity	Self-reported caste and ethnic group of respondents – Brahmin/Chhetri, Jana Jaati, Dalit, Madhesi, Muslim and others
Religion	Respondent’s self-reported religious belief at the time of survey
Education	Respondent’s reported level of educational attainment - no formal education, Primary/basic level education, secondary and higher-level education
Occupation	Respondent’s main occupation at the time of survey - labor, service, unemployed or housework, business, self-employment, farming, and cannot work
Marital status	Respondent’s self-reported marital status at the time of survey - married, ever married, single (widowed or divorced)
Disability type	Self-reported impairments and functional limitations, categorized as physical, visual, hearing, intellectual, and multiple disabilities, confirmed by their disability ID cards and or with the assessment using the WG questionnaire (short-set).
Severity of disability	Categorized as a profound, severe, moderate, and mild disability based on the government distributed red, blue, yellow, and white color disability ID cards respectively.
Household wealth index [30]	A composite measure of a household’s cumulative living standard calculated using easy-to-collect data on a household’s ownership of selected assets such as TV, cupboard, fan, materials used for housing construction - lowest, second, middle, fourth, highest

### Statistical analysis

The data collected in KoBo Collect software were downloaded into Microsoft Excel Windows 10, subsequently cleaned, and then transferred into SPSS (version 23.0 for Windows) for analysis. Before statistical analysis, a normality test was performed to determine the data skewness and kurtosis level. The test showed that the skewness for the EMIC score and P-scale data was 0.512 and 0.813, and the kurtosis was − 1.026, and − 0.325 respectively, which were found within the acceptable range between +/- 1 [31, 32]. We used both descriptive and inferential statistics to summarize the characteristics of data and determine the association between variables. Categorical variables were summarized using frequencies and percentages, and continuous variables were summarized using means and standard deviations (SDs). Independent-sample t- test and One-way analysis of variance (ANOVA) were conducted to determine the differences between groups for selected sociodemographic variables, while bivariate correlation analysis was performed to measure the strength and direction of the relationship. The relationships between perceived stigma and participation scale scores were examined using bivariate correlational analyses and multivariate linear regression models. We conducted linear regression analysis using stigma and the demographic variables as independent variables and participation score as the outcome variable to identify factors associated with participation restriction at 5% significant level. Multi-collinearity was checked with Variance Inflation Factor (VIF), which was < 2.

## Results

### Characteristics of study participants

Out of 370, a total of 369 interviews with complete information were included in the analysis. The vast majority of the participants (98%) were urban residents, and over 62% were male. The median age of the respondents was 49 (SD 18.72) years. The highest proportion of caste groups was JanaJaati (36%), followed by Brahmin and Chhetri (34%). One in twenty participants were Dalits, while Muslims and other caste and ethnic group participants accounted for less than 8%. The majority of the participants (78%) reported their religious belief as Hinduism. Over two-thirds of participants reported not having a school education. Among school-enrolled participants, just over one out of five reported primary level education, and over one in ten reported secondary or higher-level education. The highest proportion of respondents (40%) were unemployed. Among employed respondents, over one in five were in service or labor work, and approximately one in four were engaged in their own business or farm work as their main occupation. Over 16% of respondents reported that they were unable to work. Approximately 44% of respondents had physical disabilities, and 24% had hearing disabilities. Over one in ten had visual disability, while 6% had intellectual disability. Just under 15% of participants reported multiple disabilities. Just over 31% of disability ID card recipients (*n* = 210) had profound disability, while 48% had severe disability. More than half of the participants (55%) reported that they were married, more than 35% were never married, and another 10% reported their status as single (widow or divorced). More than 26% of the participants belonged to the middle-level household wealth quintile, while 17% and 21% belonged to the highest and lowest wealth quintiles, respectively. (Table 2)

### Perceived stigma

The mean EMIC score was 16.9 (SD 13.8), ranging from 0 to a maximum of 42. A total of 42% of respondents scored higher than the mean score, indicating higher levels of perceived stigma. When comparing the demographic characteristics of the participants, there were significant differences in scores between caste and ethnic groups (*P* < 0.05). Madhesi followed by Muslims had higher scores at 26.5 (SD 15.1) and 18 (SD 14.2), respectively, than other caste and ethnic groups. Brahmin and Chhetri scored the lowest at 14.1 (SD 13). Similarly, the mean differences in score among the participants having different levels of education, occupation, household wealth and disability type were statistically significant (*P* < 0.05). Participants who had no formal education had the highest mean at 18.8 (SD 13.7) and the lowest who had secondary and higher level of education at 8.3 (SD 11.5). Among the occupational groups, individuals who could not work scored highest (27.3, SD 14.9), followed by those who reported their occupation as labor (23.5, SD 14.1). Unemployed or housewife/husband had the lowest mean score at 10.9 (SD 10.1). Comparing the score between different types of disability, people with multiple disabilities had the highest mean score (23, SD 11.7), while people with physical disabilities scored the lowest (15.4, SD 14). Similarly, the lowest wealth quintile groups had the highest mean score (22.7, SD 13.9), and the middle wealth quintile groups had the lowest (14.7, SD 13.5), indicating a lower level of perceived stigma compared to the lowest wealth quintile group. The score differences were insignificant (*P* > 0.05) between the groups by place of residence, gender, age, marital status, religion, and severity of disability. (Table 2).

**Table 2:** Perceived stigma score by demographic characteristics

Variables	Frequency (%)	Mean	SD	t/F - Ratio	*P* value
Overall stigma score	369 (100)	16.9	13.8	-	-
**Place of Residence**					
Urban	361 (97.8)	16.9	13.7	-0.30	0.761
Rural	8 (2.2)	18.4	17.4		
**Gender**					
Male	230 (62.3)	15.9	13.7	-1.74	0.082
Female	139 (37.7)	18.5	13.8		
**Age in Years**					
18–24	46 (12.5)	14.6	11.5	1.00	0.409
25–34	60 (16.3)	15.9	14.8		
35–44	54 (14.6)	16.7	14.0		
45–54	63 (17.1)	16.0	12.3		
55 and above	146 (39.6)	18.5	14.5		
**Caste & Ethnicity**					
Brahmin/Chhetri	127 (34.4)	14.1	13.0	10.48	0.000
JanaJaati	133 (36.0)	15.0	12.0		
Madhesi	61 (16.5)	26.5	15.1		
Dalit	19 (5.1)	16.6	13.1		
Muslims & others	29 (7.9)	18.0	14.2		
**Religion**					
Hinduism	288 (78.0)	17.3	13.8	0.91	0.362
Others	81 (22.0)	15.7	13.5		
**Education**					
No formal education	244 (66.1)	18.8	13.7	11.76	0.000
Primary/Basic (1–8)	80 (21.7)	16.1	13.3		
Secondary and higher (Grade 9+)	45 (12.2)	8.3	11.5		
**Occupation**					
Labor	67 (18.2)	23.5	14.1	21.55	0.000
Service	15 (4.1)	21.3	14.0		
Unemployed & housework	147 (39.8)	10.9	10.1		
Business & self-employment	30 (8.1)	13.4	11.8		
Farming	49 (13.3)	14.0	10.7		
Cannot work	61 (16.5)	27.3	14.9		
**Marital status**					
Married	202 (54.7)	17.4	14.4	0.54	0.583
Never Married	130 (35.2)	16.6	12.9		
Single (Widow, Divorced)	37 (10.0)	15.0	12.9		
**Disability Type**					
Physical	162 (43.9)	15.4	14.0	3.42	0.009
Visual	42 (11.4)	16.8	12.7		
Hearing	87 (23.6)	15.9	14.5		
Intellectual	23 (6.2)	17.2	12.3		
Multiple	55 (14.9)	23.0	11.7		
**Severity of disability (** *n* ** = 210)**					
Profound	66 (31.4)	18.3	12.3	2.50	0.061
Severe	100 (47.6)	13.5	11.6		
Moderate	21 (10.0)	12.3	13.7		
Mild	23 (11.0)	16.8	14.2		
**Household Wealth Quintile**					
Lowest	77 (20.9)	22.7	13.9	4.62	0.001
Second	79 (21.4)	15.5	13.6		
Middle	98 (26.6)	14.7	13.5		
Fourth	53 (14.4)	16.7	12.9		
Highest	62 (16.8)	15.2	13.3		

### Participation restriction

Table 3 shows that the majority of participants with disabilities (56.9%) reported participation restrictions. Over one-third had severe (21.7%) and extreme (16.3%) restrictions.

**Table 3:** Grades of participation restriction

Grades	Score range	frequency	%
No significant restriction	0–12	159	43.1
Mild restriction	13–22	40	10.8
Moderate restriction	23–32	30	8.1
Severe restriction	33–52	80	21.7
Extreme restriction	53–90	60	16.3

The participation restriction differed by the demographic characteristics of the study participants. The differences between individuals’ disability types, severity, education level and occupation were statistically significant (*P* < 0.05), while place of residence, gender, age, marital status, caste and ethnicity, religion, and household wealth status were insignificant in participation restriction (*P* > 0.05). People with intellectual disabilities scored the highest mean (41.5, SD 24.2), followed by people with multiple disabilities (35.3, SD 27.1). People with hearing disabilities had the lowest mean (16.2, SD 19.1). Similarly, those with profound disability had highest mean (33.9, SD 30.4) followed by mild (25.9, SD 22.9) and severe disability (25.1, SD 22.9). Participants with no formal education had the highest mean score (30.1, SD 26), and those who reported a secondary or higher level of education had the lowest mean score (14.5, SD 17.8). Similarly, those who were unable to work and unemployed scored nearly equal at 30.7 (SD 32.9) and 30.5 (SD 24.5), respectively. Participants who reported their occupation as service scored 26.1 (SD 20.6), and those with their own business or self-employed had the lowest mean score at 18.3 (SD 19.2). (Table 4)

**Table 4:** Participation score by demographic characteristics

Variables	Frequency (%)	Mean	SD	t/F - Ratio	*P* value
**Place of Residence**					
Urban	361 (97.8)	26.5	25.1	0.35	0.730
Rural	8 (2.2)	23.4	14.4		
**Gender**					
Male	230 (62.3)	25.0	23.7	-1.35	0.177
Female	139 (37.7)	28.6	26.8		
**Age in Years**					
18–24	46 (12.5)	27.9	27.9	0.47	0.758
25–34	60 (16.3)	23.3	25.5		
35–44	54 (14.6)	27.4	25.8		
45–54	63 (17.1)	24.5	22.0		
55 and above	146 (39.6)	27.6	24.8		
**Caste & Ethnicity**					
Brahmin/Chhetri	127 (34.4)	27.7	24.7	2.39	0.050
JanaJaati	133 (36.0)	28.7	25.0		
Madhesi	61 (16.5)	17.9	25.2		
Dalit	19 (5.1)	31.5	26.9		
Muslims & others	29 (7.9)	26.7	23.7		
**Religion**					
Hinduism	288 (78.0)	26.4	25.3	0.001	0.999
Others	81 (22.0)	26.4	23.7		
**Education**					
No formal education	244 (66.1)	30.1	26.0	9.75	0.000
Primary/Basic (1–8)	80 (21.7)	21.7	22.1		
Secondary and higher (Grade 9+)	45 (12.2)	14.5	17.8		
**Occupation**					
Labor	67 (18.2)	21.5	21.6	3.01	0.011
Service	15 (4.1)	26.1	20.6		
Unemployed & housework	147 (39.8)	30.5	24.5		
Business & self-employment	30 (8.1)	18.3	19.2		
Farming	49 (13.3)	20.3	20.0		
Cannot work	61 (16.5)	30.7	32.9		
**Marital status**					
Married	202 (54.7)	25.0	23.7	2.55	0.080
Never Married	130 (35.2)	30.0	26.9		
Single (Widow, Divorced)	37 (10.0)	21.1	23.1		
**Disability Type**					
Physical	162 (43.9)	26.5	25.2	8.08	0.000
Visual	42 (11.4)	27.2	24.2		
Hearing	87 (23.6)	16.2	19.1		
Intellectual	23 (6.2)	41.5	24.2		
Multiple	55 (14.9)	35.3	27.1		
**Severity of disability (** *n* ** = 210)**					
Profound	66 (31.4)	33.9	30.4	2.70	0.047
Severe	100 (47.6)	25.1	22.9		
Moderate	21 (10.0)	18.4	18.7		
Mild	23 (11.0)	25.9	22.9		
**Household Wealth Quintile**					
Lowest	77 (20.9)	23.3	20.5	1.56	0.185
Second	79 (21.4)	26.5	26.3		
Middle	98 (26.6)	27.3	26.8		
Fourth	53 (14.4)	33.0	25.3		
Highest	62 (16.8)	23.0	24.2		

### Correlation between participation restriction, perceived stigma, and demographic characteristics

Table 5 shows the correlation between perceived stigma and participation restriction scores and their relation with sociodemographic variables. The analysis did not show any correlation between perceived stigma and participation restriction (*r* = 0.072, *P* > 0.05). However, perceived stigma and participation restriction were both found to have negative correlations with education (*r* = -0.24, *P* < 0.01; *r* = -0.21, *P* < 0.01), which means those having lower education levels had higher levels of stigma and restrictions. Additionally, perceived stigma had a positive correlation with disability type (*r* = 0.17, *P* < 0.01) and a negative correlation with severity of disability (*r* = -0.15, *P* < 0.05), and wealth index (*r* = -0.15, *P* < 0.01); however, all these correlations were weak.

**Table 5:** Correlation matrix among interest variables

Variables	Spearman’s rho correlation coefficient	
	Perceived stigma	Participation restriction
Participation restriction	0.072	
Place of residence	0.002	0.008
Gender	0.097	0.045
Age	0.093	0.051
Caste & ethnicity	-0.047	-0.018
Religion	-0.044	0.011
Education	-0.244^**^	-0.210^**^
Occupation	0.045	-0.029
Marital status	-0.026	0.017
Disability type	0.169^**^	0.067
Severity of disability	-0.149*	-0.110
Wealth index	-0.147^**^	0.001

**Correlation is significant at the 0.01 level (2-tailed).

*Correlation is significant at the 0.05 level (2-tailed).

Linear regression analysis conducted to predict factors associated with participation restriction showed that except for education (β = -9.34, *P* < 0.01), none of the demographic and other factors were statistically significant. (Table 6).

**Table 6:** Results of linear regression analysis on factors associated with participation restriction

Independent Variables	Coefficient (β)	Std. Error	t	*p*-value	VIF
Perceived stigma	0.10	0.15	0.67	0.503	1.10
Place of residence	-0.20	18.62	-0.01	0.991	1.09
Gender	3.31	3.70	0.89	0.373	1.06
Age	-1.18	1.30	-0.90	0.367	1.23
Caste and ethnicity	-0.58	1.31	-0.44	0.660	1.18
Religion	-3.82	5.15	-0.74	0.459	1.31
Education	-9.34	3.05	-3.07	0.002	1.60
Occupation	-0.12	1.20	-0.10	0.924	1.16
Marital status	-2.54	3.14	-0.81	0.419	1.23
Disability type	0.21	1.35	0.15	0.878	1.31
Severity of disability	-1.55	2.14	-0.73	0.469	1.30
Wealth Index	0.72	1.33	0.54	0.592	1.13

## Discussion

This study aimed to measure the levels of internalized stigma and participation restrictions among persons with disabilities and to assess their correlations with various sociodemographic factors. The findings revealed that almost all respondents experienced some degree of internalized stigma overall. Notably, a significant proportion (42%) reported a higher level of stigma. However, the degree of stigma varied significantly based on the type of disability and sociodemographic characteristics. Persons with multiple disabilities were found to have the greatest level of internalized stigma, while those with physical disabilities reported the lowest. This may be explained by internalized stigma among people with multiple disabilities being driven by a complex interaction between multiple factors, such as environmental, socioeconomic, cultural, psychological and others, producing a compounding effect with more negative feelings contributing to a higher level of stigma. They may have deeper feelings of self as inferior to others and a subsequent loss of status and self-esteem.

The level of stigma varied by caste/ethnicity, a person’s education level, occupation, and household wealth. The study showed that Madhesi ethnic groups, individuals with no formal education, unemployed individuals or those who cannot work and belong to the lowest wealth quintile exhibited a greater level of stigma. This indicates that the poor and more vulnerable segments of the population hold higher levels of negative feelings of self and internalization of self-devaluation. Previous studies have consistently highlighted the significant impact of enacted stigma, education status, and level of awareness in developing negative attitudes and internalization of stigma [33, 34]. Self-perception of prejudice and stigma is related to an individual’s socioeconomic and cultural environment and adaptation to a pattern of social behavior. It is commonly cited that poorer persons with disabilities face more stigma than economically advantaged persons with disabilities [35]. Additionally, studies commonly report that poor, uneducated and traditional societies associate the causes of disability with assumptions of supernatural forces or punishment from God [16]. In Nepal, such misconceptions about the causes of disability and reasons for abuse, violence and discrimination are reported as existing among the Madhesi communities [13, 36]. This could be the possible cause for the higher level of perceived stigma among Madhesi people.

This study found significant differences in perceived stigma between respondents who worked and those who were unable to work. Those who were unable to work due to their disability had significantly higher stigma scores. This may be due to the negative impact on the family’s socioeconomic status, as they have no earnings, and their full dependency on others. Consequently, this full dependency may have led to more negative attitudes and deeper feelings of self-worthlessness, contributing to a higher level of stigma. Interestingly, these findings contradict a study conducted in Indonesia, which reported no correlation between employment status (working and not working) and perceived stigma [37].

In line with the findings from several studies conducted in Asia [38] and Africa [39], this study also did not find significant gender differences in stigma scores. The findings of a systematic review on disability stigma found a neutral effect of gender on disability-related stigma [16]. In contrast, a study conducted in India reported a higher level of perceived stigma among women with psychosocial disabilities [40]. There was no effect of marital status on stigma internalization. However, we noted that 45.2% of study participants reported their marital status as unmarried, divorced or widowed. This figure is much higher compared to national statistics for the unmarried or divorced population, which is reported at 38.2% by the recent census [23]. Additionally, a study conducted in low-income countries found that 81% of unmarried (84% female and 79% male) respondents felt that their disability and/or associated stigma posed a challenge in getting married. In support of our findings, studies in South Africa and Ghana reported no association between marital status and internalized disability stigma [16]. Based on these results, it is recommended to conduct further studies to validate and reconfirm these findings.

Another important finding was the negative correlation between education and stigma score. The respondents with a higher level of education had a lower level of perceived stigma. This is consistent with findings reported from several other studies conducted in Asian and African contexts. A study in India [41] and Ethiopia [16] revealed that people with higher levels of education demonstrated lower levels of stigma. A possible explanation could be that formal education develops knowledge and intellectual maturity with information that helps educated persons understand circumstances in a better way, resulting in less stigma. As with education, perceived stigma had negative relationships with household wealth. Wealthy families may have acquired higher levels of social status and power, averting individual devaluation and self-humiliation.

The study also found a high level of participation restriction faced by a large proportion of study participants. The majority of participants (56.9%) reported that they often faced participation restrictions. Over one-third had severe or extreme restrictions. This figure is comparable with the findings from other studies in Asian contexts. Studies in Indonesia and India reported participation restrictions higher than this at 60% and 67%, respectively, and a study in China reported much lower at 46% [41–43]. Additionally, the restrictions were not uniform to all population groups. They differed according to an individual’s disability type, severity of disability, education level and household wealth status. Persons with intellectual disabilities faced the highest restriction, while those with hearing disabilities had the lowest. Individuals with profound disabilities experienced higher levels of restrictions compared to those with severe or mild disabilities. The unemployed and those who were unable to work faced more restrictions than the self-employed and those involved in farming. Education level showed a negative correlation, indicating that the higher the level of education, the lesser the restriction on participation. All of these results indicate that empowered individuals with higher socioeconomic status and higher-level education enjoy better opportunities for social activities and employment with fewer restrictions. Past studies have reported power relations with stigma and social participation that support this finding [44].

Several other studies have reported that stigma and discrimination against persons with disabilities result in feelings of domination and shame, which leads to developing withdrawal attitudes that limit individuals’ participation in the family and society [3]. However, the results of this study did not prove this hypothesis. There was no association observed between perceived disability stigma and participation restriction. This finding can be interpreted as follows: the relationships between disability stigma and participation restrictions are contextual and differ according to individual and social circumstances. This study warrants further mixed-method research to explore their relationship to reaffirm this finding.

### Limitations of the study

A number of limitations need to be considered when interpreting the findings of this study. The study design was cross-sectional; hence, it did not assess causal relationships among the variables studied. We did not include all types of disabilities, and the sample was not fully representative of all social groups. Moreover, the sample did not consider whether the disability was congenital or acquired. All these factors could have created different environmental adaptations that form the individual’s beliefs, attitudes and internalization of their stigma. Furthermore, this study did not provide the perspectives of people without disabilities or the general public; rather, it focused on the feelings and experiences of discrimination and restrictions faced by persons with disabilities. Further research should be conducted in diverse settings using samples with different types, levels, and severity of disability, including community perspectives, to better understand the stigma, participation restriction and oppression faced by persons with disabilities.

## Conclusion

This study found that persons with disabilities carried some degree of perceived stigma in general; however, a substantial proportion of them had higher levels of stigma. The magnitude of stigma varied depending on the type of disability, the severity of impairment, the person’s education level, occupation, and sociocultural environment. Notably, there was a negative correlation between perceived stigma and education level, as well as household wealth. People with multiple disabilities were particularly prone to high levels of perceived stigma. Additionally, the findings of this study also revealed that 56.9% of sample respondents had confronted notable participation restrictions. The extent of these restrictions varied among different groups. Persons with intellectual disabilities, those who had profound or severe disability, unemployed or unable to work, and individuals without formal education were found to face the highest levels of participation restrictions. Education and participation restriction had a negative association. However, no relationship was found between perceived stigma and participation restrictions. To address these challenges, it is crucial to implement stigma reduction programs that prioritize education and empowerment. Such programs can play a significant role in overcoming internalized stigma and promoting greater participation of persons with disabilities.

## Data Availability

The datasets used and/or analyzed during the current study are available from the corresponding author on reasonable request.

## Abbreviations

ANOVA: Analysis of variance
EMIC: Explanatory Model Interview Catalog
ICF: International Classification of Functioning
OPD: Organization of Persons with Disabilities
P-Scale: Participation Scale
PwD: Person with Disability
SD: Standard deviation
UNCRPD: United Nations Convention on the Rights of Persons with Disabilities
